# On the Counterfactual Nature of Gambling Near‐misses: An Experimental Study

**DOI:** 10.1002/bdm.2010

**Published:** 2017-04-03

**Authors:** Yin Wu, Eric van Dijk, Hong Li, Michael Aitken, Luke Clark

**Affiliations:** ^1^ Research Center for Brain Function and Psychological Science Shenzhen University Shenzhen China; ^2^ Behavioural and Clinical Neuroscience Institute, Department of Psychology University of Cambridge Cambridge UK; ^3^ Department of Social and Organizational Psychology and Leiden Institute for Brain and Cognition Leiden University Leiden The Netherlands; ^4^ Shenzhen Institute of Neuroscience Shenzhen China; ^5^ Department of Psychology Institute of Psychiatry, Psychology and Neuroscience, King's College London London UK; ^6^ Centre for Gambling Research at UBC, Department of Psychology University of British Columbia Vancouver British Columbia Canada

**Keywords:** near‐misses, counterfactual thinking, luck, reflection and evaluation model, cognitive distortions

## Abstract

Research on gambling near‐misses has shown that objectively equivalent outcomes can yield divergent emotional and motivational responses. The subjective processing of gambling outcomes is affected substantially by close but non‐obtained outcomes (i.e. counterfactuals). In the current paper, we investigate how different types of near‐misses influence self‐perceived luck and subsequent betting behavior in a wheel‐of‐fortune task. We investigate the counterfactual mechanism of these effects by testing the relationship with a second task measuring regret/relief processing. Across two experiments (Experiment 1, *n* = 51; Experiment 2, *n* = 104), we demonstrate that near‐wins (neutral outcomes that are close to a jackpot) decreased self‐perceived luck, whereas near‐losses (neutral outcomes that are close to a major penalty) increased luck ratings. The effects of near‐misses varied by near‐miss *position* (i.e. whether the spinner stopped just short of, or passed through, the counterfactual outcome), consistent with established distinctions between upward versus downward, and additive versus subtractive, counterfactual thinking. In Experiment 1, individuals who showed stronger counterfactual processing on the regret/relief task were more responsive to near‐wins and near‐losses on the wheel‐of‐fortune task. The effect of near‐miss position was attenuated when the anticipatory phase (i.e. the spin and deceleration) was removed in Experiment 2. Further differences were observed within the objective gains and losses, between “clear” and “narrow” outcomes. Taken together, these results help substantiate the counterfactual mechanism of near‐misses. © 2017 The Authors Journal of Behavioral Decision Making Published by John Wiley & Sons Ltd.

## Introduction

The outcomes of decisions we make have a pronounced effect upon our emotional state: we feel happy after obtained successes, and sad or disappointed following losses and defeats. This focus on factual outcomes fits the assumption of traditional economic theory that we wish to maximize the outcomes we obtain (Kahneman, 2011). However, it is increasingly recognized that our feelings are also influenced by “counterfactual outcomes”: outcomes we could have obtained if only reality had taken another turn. Research on counterfactual thinking shows that we are often strongly affected by what might have happened, or what nearly happened. An anecdote by Kahneman and Tversky (1982) described two travelers, one who missed his flight by 5 minutes, and the other who missed the same flight by 30 minutes. Objectively, these two outcomes are equivalent in that neither traveler caught the plane, but 96% of participants expected the first traveler to feel worse. Here, the closeness to the desired outcome creates an upward counterfactual (“He almost made the flight!”), such that a narrowly missed desirable outcome intensifies the emotional response (in this case, regret).

A more extreme example arises when people who perform objectively better in a contest can ultimately feel worse than those who perform less well, a phenomenon termed “satisfaction reversal” (Medvec & Savitsky, 1997). Olympic silver medalists describe less satisfaction at their achievements than bronze medalists (Medvec, Madey, & Gilovich, 1995), presumably due to the opposite influences of the counterfactual thoughts “I nearly won the gold” (silver) and “I nearly missed out on a medal” (bronze). Medvec and Savitsky (1997) developed these observations into a model of categorical cutoff points: values that impose qualitative boundaries on quantitative outcomes (which are frequently arbitrary, such as exam thresholds) can thereby induce counterfactual thoughts. As part of their model, they showed that the simple act of surpassing a grade cutoff elicits downward counterfactuals and increases positive affect, and conversely just missing a cutoff triggers upward counterfactuals and decreased satisfaction.

These effects of counterfactual outcomes are ubiquitous in gambling behavior, which itself offers a paradigm for studying decision‐making more broadly (see Clark et al., 2013, for a review). The classic gambling “near‐miss” refers to a non‐win outcome that falls tantalizingly close to the jackpot (Clark, Lawrence, Astley‐Jones, & Gray, 2009; Reid, 1986), such as a horse finishing in second place in a neck‐to‐neck finish. Previous research has shown that these events (henceforth labeled “near‐wins” (NW) to avoid any semantic confusion between nearly winning and nearly losing) are experienced as aversive, but increased motivations to continue gambling (Clark, Crooks, Clarke, Aitken, & Dunn, 2012; Clark et al., 2009). Recent studies have extended the near‐miss phenomenon into the loss domain, enabling a comparison between NW and near‐losses (NL). Experiments measuring subjective responses to neutral outcomes that were close to jackpot (i.e. NW) as well as neutral outcomes that were close to a major penalty (i.e. NL) indicate that NL are also processed as a discrete class of event (Dillon & Tinsley, 2008; Wohl & Enzle, 2003; Wu, van Dijk, & Clark, 2015; Zhang & Covey, 2014).

Past work on these types of decision outcomes has tended to focus on anecdotal scenarios (Dillon & Tinsley, 2008) or single‐shot decisions (Wohl & Enzle, 2003). In the present study, we developed a multi‐shot task based on a wheel‐of‐fortune game to compare responses to these various outcome types within the same participant. We looked at how these outcomes influenced perceptions of luck, and betting decisions on the subsequent trial. Luck ratings capture the element of chance in decision outcomes and are known to be sensitive not only to the objective outcome valence, but also to close counterfactuals (Teigen, 1995). We also measured trial‐by‐trial bet amount change as a function of the preceding trial (see also Demaree, Burns, Dedonno, Agarwala, & Everhart, 2012).

**Figure 1:**
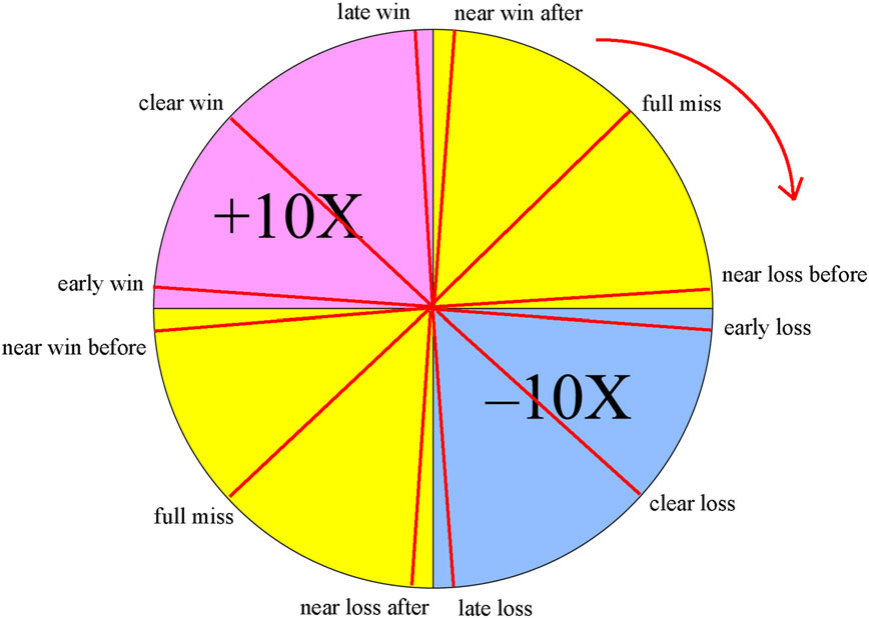
The wheel‐of‐fortune task. The arrow outside of the wheel indicates the movement direction of the spinner. [Colour figure can be viewed at wileyonlinelibrary.com]

Our first aim was to investigate how people respond to null outcomes that differed only in whether they were close to a win (i.e. NW) or close to a loss (i.e. NL) (see Figure 1). In a prior study using a wheel‐of‐fortune task where we highlighted each wheel segment successively, we found that null outcomes that were close to a significant penalty elicited downward counterfactuals and increased self‐perceived luck, whereas null outcomes that were close to a jackpot elicited upward counterfactuals and decreased self‐perceived luck (Wu, van Dijk, Clark, 2015; see also Wohl & Enzle, 2003). We sought to corroborate these effects of NW and NL using an improved version of the task with a spinner that allowed a continuously varying position, so that outcomes could fall close to the boundary to the next segment.

The second aim was to explore if these effects of near outcomes were further moderated by their position relative to the missed outcome; that is to say, when the spinner stopped just *before* the win/loss segment, compared to when the spinner stopped just *after* the win/loss segment. Using a slot machine task, we have previously described how NW that stop just short of the winning line primarily act to increase motivation to continue, whereas NW that pass through the winning line generate a more aversive effect (Clark, Liu, et al., 2013). These differential effects can be explained in terms of counterfactual thinking, drawing upon the distinction between additive and subtractive counterfactual thoughts. Additive counterfactuals refer to those that add hypothetical events to reality (e.g. “If only I had an umbrella, I would not have gotten wet”), whereas subtractive counterfactuals involve removing or “undoing” events from reality (i.e. “If only it hadn't rained today, I would not have gotten wet”) (Roese & Olson, 1993). Additive and subtractive counterfactuals have differential effects upon mood and behavioral regulation (Roese, 1994). On a slot machine, NW where the reel stops before the payline position would likely generate a counterfactual thought that the reel's trajectory might have continued to the jackpot position (an additive counterfactual), whereas for the NW after the payline, the gambler must mentally reverse the subsequent step, a subtractive counterfactual (Clark et al., 2013). This difference in the type of counterfactual thought may explain the contrasting emotional and motivational effects engendered by these two events (Clark et al., 2013; see also Markman & McMullen, 2003).

In the present study (Experiment 1 and 2), the spinner decelerated in a clockwise direction, and it could stop fractionally before entering a winning (or losing) segment, or just after exiting a winning (or losing) segment. Based upon the slot machine data, we predicted that the spinner stopping just after a win location (NW after, henceforth a “NW‐after”) would be perceived as unluckier than when it stopped just before (henceforth a “NW‐before”), and that a NW‐before may increase the subsequent amount bet as a reflection of increased motivation.

The other major aim of this study was to examine the relationship between individual differences in reactions to near‐miss events, and the behavior on a second task assessing regret and relief processing (Mellers, Schwartz, & Ritov, 1999). Prior work using anecdotes has asked participants to endorse counterfactual statements (Medvec et al., 1995; Medvec & Savitsky, 1997) or to reflect on “how things could be different” (Wohl & Enzle, 2003). These studies illustrate that narrowly missing more or less desirable outcomes elicited regret or relief (respectively), but these methods may be considered prone to demand characteristics. Other research on NW has primarily described these events as triggering frustrative non‐reward (Wadhwa & Kim, 2015) or attributions of skill acquisition (Clark et al., 2013), mechanisms that need not inherently rely on counterfactual processing. In the present study, we sought to test the link between gambling near‐misses and counterfactual thinking using a different approach, looking at individual differences in “counterfactual potency” on an independent task (Camille et al., 2004; Camille et al., 2010; Gillan et al., 2014; Wu & Clark, 2015). Previous research has characterized counterfactual potency as the multiplicative combination of “if likelihood” and “then likelihood”, and showed this parameter correlated with intensity of emotional responses (Petrocelli, Percy, Sherman, & Tormala, 2011). In Experiment 1, we used a second decision‐making task where participants choose between two gambles, and after viewing their obtained outcome, the non‐obtained outcome from the unselected gamble was displayed. In this task, affect ratings taken after each trial are sensitive not only to size of the obtained outcome, but also to the non‐obtained outcome. For example, negative affect is strongest when the obtained outcome is a loss *and* the non‐obtained outcome is revealed to be a major win (Camille et al., 2004, 2010; Gillan et al., 2014; Mellers et al., 1999). We quantified counterfactual potency as the slope of function for affect ratings based upon the difference between obtained and non‐obtained outcomes, such that steeper slopes indicate greater modulation by the difference between the outcomes. We analyzed the correlation between this index and the luck ratings following near events on the wheel‐of‐fortune task, predicting that participants with higher counterfactual potency would be more sensitive to near‐misses.

Our task also enabled a more exploratory analysis decomposing the analogous subtypes of objective wins and losses. In the present study, the spinner could stop either centrally in the win (or loss) segment—a *clear* outcome—or near the boundary to the adjacent null segment, a *narrow* outcome. These events are commonplace within both gambling games (e.g. winning a horserace by a clear distance or a neck‐to‐neck finish) and other aspects of daily life (e.g. making it to the airport with 2 hours to spare, or 5 minutes). In a stock market simulation (Markman & Tetlock, 2000), participants gave more negative ratings when their chosen stock outperformed the un‐chosen stock by a narrow margin (henceforth a “narrow win”) compared to when the chosen stock substantially outperformed the other stock (henceforth a “clear win”). These effects were mirrored for losses, and outcome closeness further impacted subsequent willingness to invest. In an analysis of NBA basketball games, teams that were losing by narrow margin at half‐time increased their effort and were ultimately more likely to win the match, compared to teams that were winning by narrow margin at the interval (Berger & Pope, 2011). Similar to the processing of NW and NL, these responses to clear versus narrow wins/losses likely also involve counterfactual thinking (Markman & Tetlock, 2000). We hypothesized that narrow wins compared to clear wins would prompt downward counterfactuals and make our participants feel luckier, and conversely narrow losses compared to clear losses would elicit upward counterfactuals and make people feel unluckier. These analyses further considered the narrow event position, distinguishing early events that have just entered the win/loss segment (henceforth a “early win” or “early loss”) against late events that have almost left the win/loss segment (henceforth a “late win” or “late loss”).

## Experiment 1

### Methods

#### Participants

We recruited 51 healthy volunteers (26 men, mean age = 24.69, *SD* = 4.16, age range = 19 – 35) from the student population at the University of Cambridge for a study of gambling behavior. Our advert stated that participants should be psychiatrically healthy, and it was directed toward students with some interest in gambling (“Do you enjoy gambling?”). We excluded psychology and economics students. The Problem Gambling Severity Index (Ferris & Wynne, 2001) was administered to screen for potential gambling problems. No participants were classified as problem gambler (a score of 8 or more), and the majority of participants (61%) scored 0. The study was conducted in accordance with Declaration of Helsinki and was approved by the University of Cambridge Psychology Research Ethics Committee. Written informed consent was obtained from all participants. Volunteers attended an individual testing session, which comprised the wheel‐of‐fortune task and the counterfactual thinking task. They were paid a fixed fee as reimbursement for their time, plus a financial bonus that was proportional to their actual earnings in the gambling tasks. Additional psychophysiological data collected in this sample have been reported elsewhere with different purposes (Wu & Clark, 2015; Wu, van Dijk, Aitken, and Clark, 2016).

#### Wheel‐of‐fortune task

Participants completed 76 experimental trials on a computerized wheel‐of‐fortune task modified from Wu, van Dijk, and Clark (2015), using a spinner rather than highlighted segments to indicate gambling outcome. The task was programmed in Matlab, using the Psychophysics Toolbox extensions (Brainard, 1997). On each trial, the wheel was divided into four segments of different colors. The “+” or “−” symbols in each segment indicated the amounts stood to win or lose. Those segments without any symbols represented zero outcomes (neither win nor lose). The number (e.g. 10) indicated the size of win/loss, as a multiplier of the amount participants bet on that round. For instance, +10× meant that the participant would win 10 times the bet, and −10× would lose 10 times of the bet.

**Figure 2:**
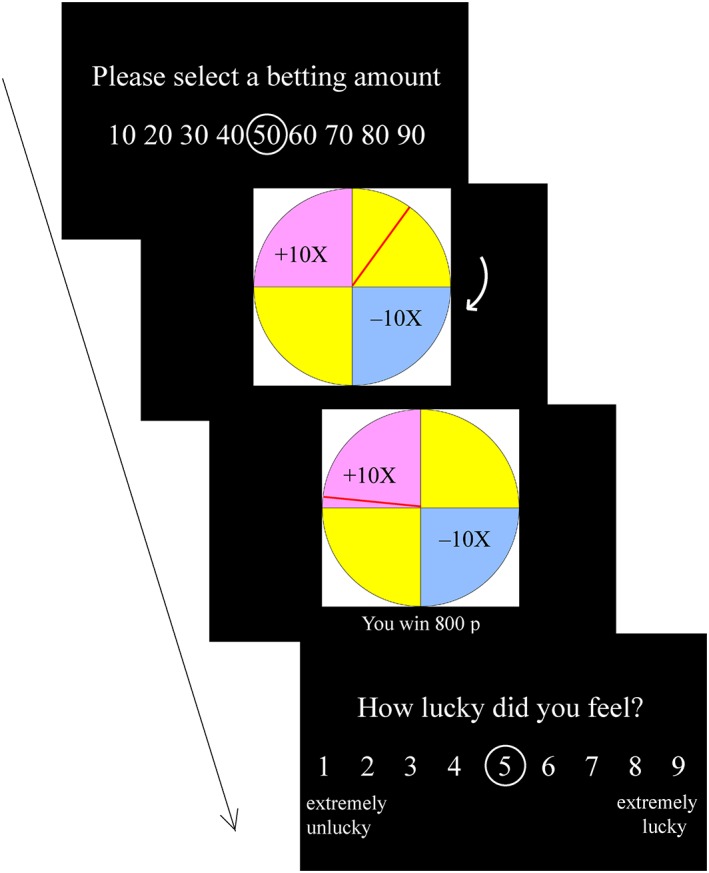
Trial timing for the wheel‐of‐fortune task. [Colour figure can be viewed at wileyonlinelibrary.com]

The trial sequence and timings are displayed in Figure 2. At the beginning of each trial, the participant was asked to choose a bet between £0.10 and £0.90, in £0.10 increments. Following bet selection, the spinner on the wheel spun for an anticipation interval (5.3 – 6.9 seconds), during which time the spinner decelerated to a standstill. The outcome phase then lasted 1 second, where the spinner stopped, and there was accompanying auditory feedback (applause sound for winning outcomes, booing sound for losing outcome and thud sound for null outcome), and the numeric outcome was displayed for 1 second. Following the outcome phase, a luck rating was displayed using a 9‐point visual analogue scale (“How lucky did you feel?”), with 1 indicating “extremely unlucky” and 9 indicating “extremely lucky”. No time constraints were imposed on the bet selection or luck rating.

The outcomes were fair, with each event type repeated five times. The closeness was manipulated in such a way that on the near event trials, the distance of the spinner to the segment boundary was 1.8°. For the clear outcomes (i.e. clear‐wins, clear‐losses and two types of full‐misses (FM)), the spinner stopped 45° from the boundary of the segment. We interspersed 16 filler trials where the spinner landed at various other positions on the wheel in order to make the task more realistic. On average, participants won £9.59 (*SD* = 16.43) in this task.

#### Counterfactual thinking task

**Figure 3:**
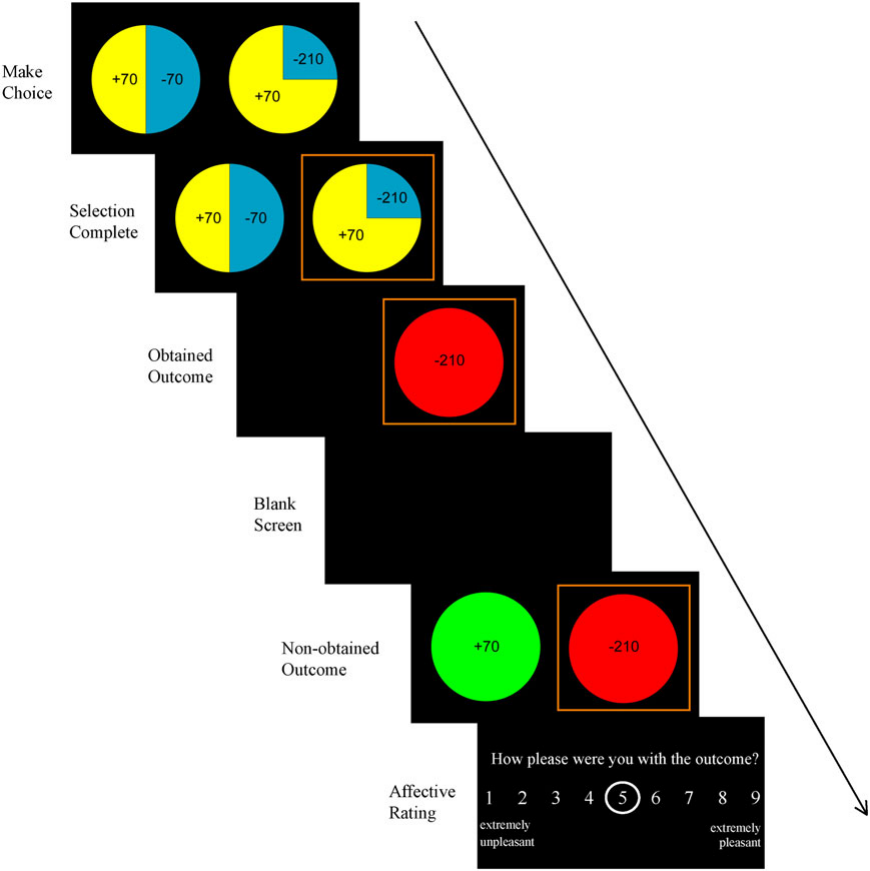
Trial timing for the counterfactual thinking task. [Colour figure can be viewed at wileyonlinelibrary.com]

Following the wheel‐of‐fortune task, participants completed a counterfactual thinking task adapted from Camille et al. (2004) (see Wu & Clark, 2015 for analysis of facial muscle responses on this task). On each of 112 trials, participants chose between two wheels that displayed different potential gains and losses, and their respective probabilities. Each wheel offered two of the following possible outcomes: +70, +210, −70, −210, representing monetary values in pence (i.e. British £). Outcome probability was illustrated by the segment size occupied by that outcome (0.25, 0.5 or 0.75, see Figure 3). As the participant selected a wheel, the wheel was highlighted with a red surround. The obtained outcome on that wheel was presented for 4 seconds, during which time the non‐selected wheel was hidden. After a further 4 seconds of blank screen, the outcome on the non‐selected wheel (i.e. the non‐obtained outcome) was revealed for 4 seconds. Participants were then asked to rate “how pleased were you with the outcome”, with 1 indicating extremely unpleasant and 9 indicating extremely pleasant. This was followed by a 4 second inter‐trial interval (to optimize the task for psychophysiology, not reported here). No time constraints were imposed on wheel selection or affect ratings. Outcomes were pre‐specified in line with the displayed probabilities in order that the task was fair. On average, participants won £12.65 (*SD* = 5.51) on the task.

#### Statistical analysis

Wheel‐of‐fortune task: We used R and *nlme* (Pinheiro, Bates, DebRoy, & Sarkar, 2013) to perform two linear mixed effects analyses on the dependent variables: (i) luck ratings (centered; 0 means neither lucky nor unlucky); (ii) the change in the bet amount (from the current *n* trial to the next, *n* + 1, trial). We use linear mixed‐effects (LME) modeling via restricted maximum likelihood for all repeated‐measures analyses (Judd, Westfall, & Kenny, 2012). As a random effect, we had an intercept representing participant number. For the two dependent variables, we ran a series of LME models to test each set of hypotheses. In a preliminary model run as a manipulation check, we assessed the impact of the *objective* outcomes (e.g. wins, losses and null) as a fixed effect. In the second step, we compared three types of null outcomes, i.e. NW, NL and FM. In the third step, we considered whether near‐miss position (before vs. after) was relevant, treating both near‐miss type and position as fixed effects (with interaction terms). In the final step, we compared the three types of win outcomes (model 4a, i.e. early‐wins, clear‐wins and late‐wins), and the three types of loss outcomes (model 4b, i.e. early‐losses, clear‐losses and late‐losses). Visual inspection of residual plots did not reveal any obvious deviation from homoscedasticity or normality. For the models on luck ratings, the bet amount at the start of current trial (i.e. before the outcome was delivered) was entered as a fixed factor of no interest. To assess the validity of the mixed effect analysis, we performed likelihood ratio tests comparing the models with fixed effects to the null models with only the random effects. We rejected results in which the model including fixed effects did not differ significantly from the null model.

Counterfactual thinking task: For the affect ratings following the non‐obtained outcomes, the size of the obtained and non‐obtained outcomes were entered as predictors, along with the interaction term. The counterfactual index was calculated by regressing the difference between what was obtained and what could have been obtained had the participant chosen the other wheel (obtained outcome minus non‐obtained outcome on the non‐selected wheel) against the subjective ratings. A steeper slope (i.e. more positive value) indicated greater relief for downward counterfactuals and stronger regret for upward counterfactuals.

### Results and discussion

#### Wheel‐of‐fortune task

##### Objective outcomes

###### Luck ratings

**Figure 4:**
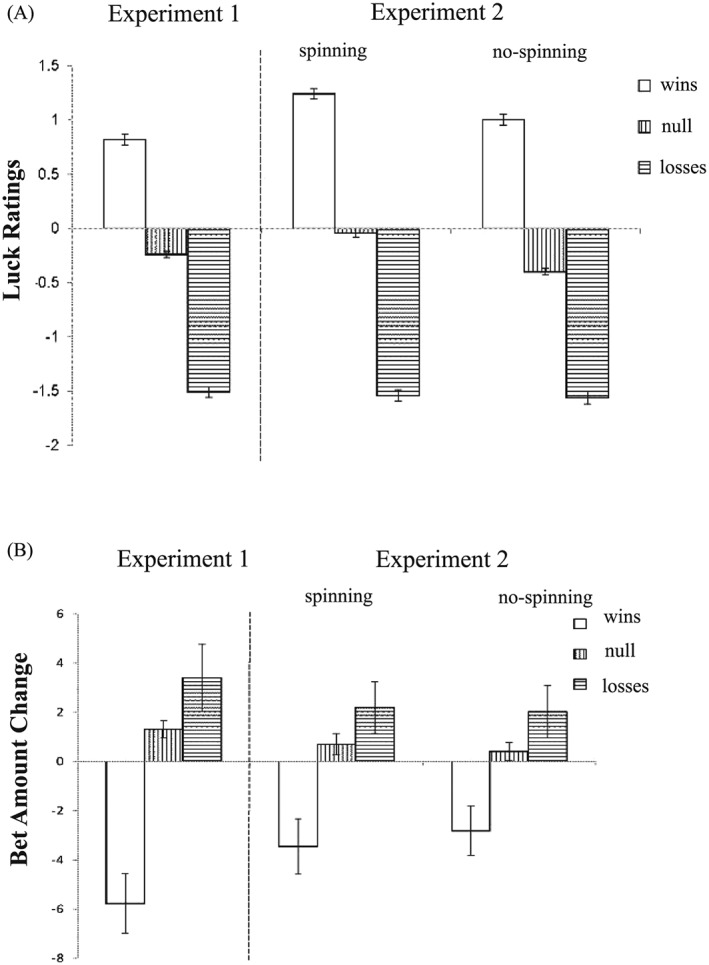
(A) Luck ratings following the three types of objective outcomes and (B) bet amount change following the three types of objective outcomes. Error bars represent standard errors of the mean

The first model investigated the effect of the different objective outcomes (three levels: wins vs. losses vs. neutral) on luck ratings (see Figure 4A). There was a significant main effect of Outcome Type, *χ^2^*(2) = 117.65, *p* < .001, with participants feeling luckier following wins compared to neutral outcomes, *b* = 1.05, *t*(100) = 6.64, *p* < .001, and following neutral outcomes compared to losses, *b* = 1.27, *t*(100) = 8.01, *p* < .001.

###### Betting behavior

The objective outcomes also impacted differently upon betting behavior (see Figure 4B), *χ^2^*(2) = 36.11, *p* < .001, with participants reducing their bet following wins compared to both neutral outcomes, *b* = −7.09, *t*(100) = −4.66, *p* < .001, and losses, *b* = −9.17, *t*(100) = −6.02, *p* < .001. There was no statistical difference between losses and neutral outcomes on bet amount change, *b* = 2.08, *t*(100) = 1.37, *p* = .18.

Thus, as a manipulation check, our participants felt luckier following wins and unluckier following losses, confirming that the task effectively induced distinct luck perceptions for the basic objective outcomes. The finding that the amount bet reduced following wins is consistent with a broad definition of the “gambler's fallacy” that people do not expect runs to continue in a random sequence (Ayton & Fischer, 2004).

##### Decomposing neutral outcomes

###### Luck ratings

**Figure 5:**
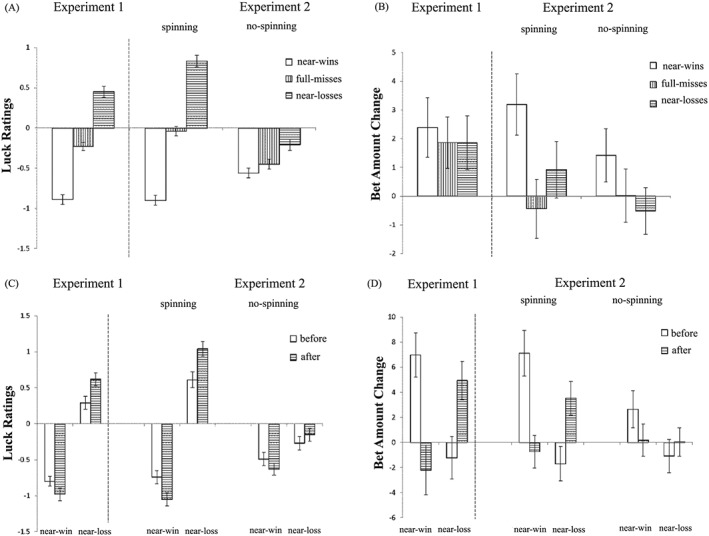
(A) Luck ratings following the three types of null outcomes, (B) bet amount change following the three types of null outcomes, (C) luck ratings as a function of Near‐Miss Type (Near‐Win, Near Loss) and Near‐Miss Position (Before, After) and (D) bet amount change as a function of Near‐Miss Type (Near‐Win, Near Loss) and Near‐Miss Position (Before, After). Error bars represent standard errors of the mean

In the next set of tests, we compared the three types of neutral outcomes (see Figure 5A), i.e. NW versus NL versus FM. There was a significant main effect of Outcome Type, *χ^2^*(2) = 81.89, *p* < .001. NL significantly increased luck ratings compared to FM, *b* = 0.68, *t*(100) = 5.70, *p* < .001, while NW significantly reduced luck ratings relative to FM, *b* = −0.66, *t*(100) = −5.50, *p* < .001.

###### Betting behavior

There was no difference in betting behavior following the different types of neutral outcomes (see Figure 5B), *χ^2^*(2) = .21, *p* > .1.

##### Near outcomes by position

###### Luck ratings

The third model distinguished four types of near‐misses based on both near‐miss type (NW vs. NL) and near‐miss position (before vs. after) (see Figure 5C). The interaction term was significant, *χ^2^*(1) = 9.90, *p* = .001. For NW, the NW‐after were rated as unluckier than NW‐before, *b* = 0.19, *t*(50) = 2.27, *p* < .05. For NL, NL‐after were rated as luckier than NL‐before, *b* = −0.41, *t*(50) = −3.24, *p* < .01.

###### Betting behavior

While we observed no overall effect of near‐misses on betting in the previous model, a significant interaction was observed between near‐miss type (NW vs. NL) and near‐miss position (before vs. after) on betting behavior (see Figure 5D), *χ^2^*(1) = 18.84, *p* < .001. Following NW, participants reduced their bet for NW‐after compared to NW‐before, *b* = 9.18, *t*(50) = 3.46, *p* = .001. Following NL, participants increased their bet following NL‐after compared to NL‐before, *b* = 6.16, *t*(50) = 2.72, *p* < .01.

Thus, NW decreased self‐perceived luck, whereas NL increased self‐perceived luck. This effect was moderated by near‐miss position, such that NW‐after were perceived as unluckier than NW‐before, consistent with the previous observation that an aversive response was stronger with NW‐after (Clark et al., 2013). On betting behavior, NW‐before increased subsequent bet amount compared to NW‐after, replicating the motivational effect of NW in the slot machine task (Clark et al., 2013, 2009; Qi, Ding, Song, & Yang, 2011). Conversely, NL‐after was rated as significantly luckier than NL‐before, and NL‐after increased bet amount more than NL‐before.

##### Subtypes of objective wins

###### Luck ratings

**Figure 6:**
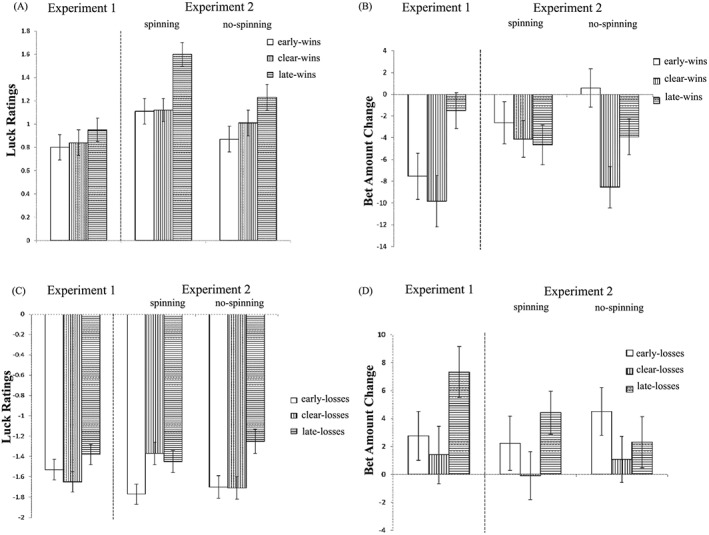
(A) Luck ratings following the three types of objective wins, (B) bet amount change following the three types of objective wins, (C) luck ratings following the three types of objective losses and (D) bet amount change following the three types of objective losses. Error bars represent standard errors of the mean

In the next step, we distinguished the three types of win outcomes, i.e. early‐wins versus clear‐wins versus late‐wins (see Figure 6A). There was a significant main effect of Outcome Type, *χ^2^*(2) = 7.52, *p* < .05, with late‐wins increasing luck feelings compared to clear‐wins, *b* = 0.30, *t*(100) = 2.77, *p* < .01. No difference between early‐wins and clear‐wins was found, *b* = 0.13, *t*(100) = 1.15, *p* > .1.

###### Betting behavior

The three subtypes of win outcomes also exerted a differential effect on the subsequent bet amount (see Figure 6B), *χ^2^*(2) = 8.88, *p* = .01, with the overall reduction in betting seen most strongly for early‐wins and clear‐wins, relative to late‐wins, *b* = −6.04, *t*(100) = −2.11, *p* < .05, and *b* = −8.35, *t*(100) = 2.92, *p* < .01, respectively.

##### Subtypes of objective losses

###### Luck ratings

In distinguishing the three subtypes of losses, i.e. early‐losses versus clear‐losses versus late‐losses (see Figure 6C), the main effect of Outcome Type was at a trend level of significance, *χ^2^*(2) = 5.52, *p* = .06, and should thus be interpreted with caution. In the pairwise comparisons, clear‐losses were rated significantly unluckier than late‐losses, *b* = −0.22, *t*(100) = 2.32, *p* = .02.

###### Betting behavior

For the differential effect of loss type on bet amount change, there was a significant main effect of Outcome Type (see Figure 6D), *χ^2^*(2) = 9.50, *p* < .01, with late‐losses increasing bet amount compared to both early‐losses and clear‐losses, *b* = 4.56, *t*(100) = 2.30, *p* < .05, and *b* = 5.92, *t*(100) = 2.98, *p* < .01, respectively.

Thus, betting behavior differed between clear‐cut and close‐call outcomes for objective gains and losses, with marginal evidence for an analogous effect on luck ratings. For objective wins, late‐wins (one type of narrow‐wins) were perceived as luckiest, and this appeared to attenuate the reduction in bet amount following wins. For objective losses, late‐losses (one type of narrow‐loss) were rated as luckiest, and elicited the largest increase in subsequent betting following losses.

##### Counterfactual thinking task

Affect ratings following the presentation of non‐obtained outcomes were first analyzed using the magnitude of the obtained and non‐obtained outcomes as two predictors, as well as their interaction term. Importantly, the affect ratings were modulated by the non‐obtained outcome, such that the participants felt worse when the non‐obtained outcome was more positive (i.e. regret) and reported higher affect when the non‐obtained outcome was more negative (i.e. relief), *b* = −0.0057, *t* = −40.38, *p* < .001. This confirms that the task effectively induced counterfactual comparisons. There was also an expected main effect of obtained outcome, as well as a significant interaction effect. 1We decomposed the interaction effect by looking at the effect of non‐obtained outcomes at each level of objective outcome. When participants objectively won the maximum amount (i.e. +210), they felt subjectively better if the non‐obtained outcomes were more negative (i.e. relief), *b* = −0.002, *t* = −4.39, *p* < .001. When they objectively won a moderate amount (i.e. +70), they felt worse if the non‐obtained outcome was more positive (+210, i.e. regret) and felt better if the non‐obtained outcomes were more negative (−70 or −210, i.e. relief), *b* = −0.005, *t* = −32.55, *p* < .001. This slope was steepest when participants objectively lost a moderate amount (i.e. −70), *b* = −0.007, *t* = −36.27, *p* < .001. When participants lost the maximum amount (i.e. −210), they felt worse if the non‐obtained outcomes were more positive (i.e. regret), *b* = −0.005, *t* = −3.88, *p* < .001, but this effect was somewhat blunted by a floor effect (see Wu & Clark, 2015).


**Figure 7:**
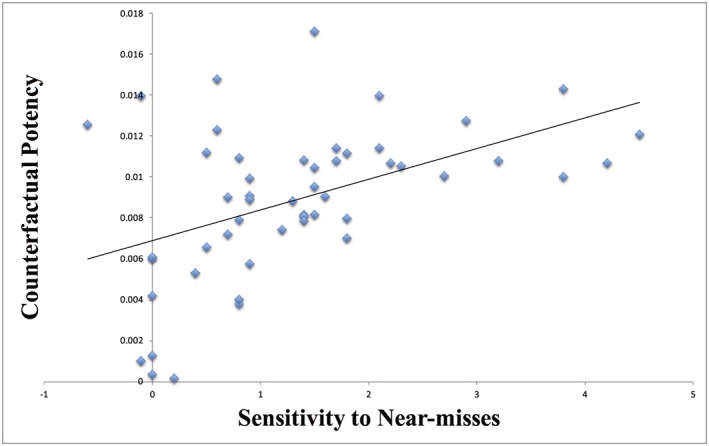
Correlation between individual's sensitivity to near‐misses and counterfactual thinking. [Colour figure can be viewed at wileyonlinelibrary.com]

The slope of the affect ratings as a function of the difference between the obtained and non‐obtained outcomes was used to index counterfactual potency: a more positive value indicates greater sensitivity to regret and relief. The change of luck ratings from NW to NL (i.e. NL – NW) in the wheel‐of‐fortune task provided an index of sensitivity to near‐misses (more positive values indicate greater responsivity to near‐misses). Across individuals, the slope of the regression line in the counterfactual thinking task was *positively* correlated with the change score in the wheel‐of‐fortune task (see Figure 7), *r* = 0.47, *p* < .001. We also assessed the relationships between counterfactual potency and sensitivity to the subtypes of near‐misses (see Table 1). Counterfactual potency was *negatively* correlated with NW‐before (i.e. NW‐before—FM, more negative values indicate greater responsivity, because NW‐before decreased luck ratings compared to FM), *r* = −0.36, *p* < .01, and NW‐after (i.e. NW‐after—FM), *r* = −0.47, *p* < .001. Counterfactual potency was *positively* correlated with NL‐before (i.e. NL‐before—FM: more positive values indicate greater responsivity, because NL‐before increased luck ratings compared to FM), *r* = 0.34, *p* = .015.

**Table 1:** Correlation between counterfactual potency and each subtype of outcome

	*r*	*p*
Near‐win before	−0.36	.0086
Near‐win after	−0.47	.0006
Near‐loss before	0.34	.0151
Near‐loss after	0.24	.0908
Early win	−0.24	.0865
Late win	−0.11	.4418
Early loss	−0.02	.8778
Late loss	0.09	.5499

## Experiment 2

The correlation between near‐miss and regret/relief tasks provides evidence for a counterfactual mechanism for near‐misses. The position effect observed on the near‐miss task is also consistent with a counterfactual account, based on additive versus subtractive thoughts. This counterfactual explanation for the position effect assumes a mental simulation that may be elicited by the motion trajectory of the spinner during the anticipation phase. However, other aspects of outcome processing may not require the visual input of the motion trajectory and depend only upon the final outcome. For example, participants' prior experience with spinning wheels may be sufficient to simulate the basic difference between NW, NL, and the objective gains and losses; the movement leading up to those outcomes may be psychologically irrelevant. For Experiment 2, we reasoned that a manipulation of the anticipatory phase would provide a further test of the counterfactual hypothesis. We compared two groups of participants: the first group played an identical wheel‐of‐fortune task as in Experiment 1. The second group performed the task with one modification: we removed the visual presentation of the wheel spin and deceleration, thus removing any visual influence of the motion trajectory in generating the near‐miss position effect. We hypothesized that if the near‐miss position effect depends on the motion trajectory, then the distinction between near‐misses in the “before” and “after” positions should disappear in the modified (“no‐spin”) version. We also aimed to corroborate the main findings of Experiment 1 in a separate group of participants.

### Methods

One hundred and four healthy Chinese students (51 men, mean age = 21.56, *SD* = 1.44, age range = 19 – 26) were randomized to two conditions of the wheel‐of‐fortune task. No participants were classified as problem gamblers based on a translated Chinese version of Problem Gambling Severity Index. For the spinning version (*n* = 52), the task was identical to Experiment 1. For the no‐spin version (*n* = 52), following bet selection, the participants viewed a 4 second blank screen followed by a reveal of the final position of the spinner on the wheel, indicating the outcome for that trial. For data analysis, Condition (spinning vs. no‐spinning) was added as a between‐subjects factor.

### Results and discussion

#### Objective outcomes

##### Luck ratings

In the first model investigating the objective outcomes (wins vs. losses vs. neutral) in the two Conditions (spin vs. no spin), the main effect of Outcome Type was significant (see Figure 4A), *χ^2^*(2) = 287.54, *p* < .001, with participants feeling luckier following wins compared to neutral outcomes, *b* = 1.34, *t*(202) = 12.30, *p* < .001, and following neutral outcomes compared to losses, *b* = 1.34, *t*(202) = 12.14, *p* < .001. There was no significant main effect of Condition, *χ^2^*(1) = 0.28, *p* > .1, or Outcome Type × Condition interaction, *χ^2^*(2) = 2.06, *p* > .1.

##### Betting behavior

There was a significant main effect of Outcome Type (see Figure 4B), *χ^2^*(2) = 34.80, *p* < .001. Wins reduced betting compared to both losses and neutral outcomes, *b* = −5.25, *t*(202) = −5.88, *p* < .001, and *b* = −3.70, *t*(202) = −4.14, *p* < .001, respectively. The difference between losses and neutral outcomes was only marginally significant, *b* = 1.56, *t*(202) = 1.74, *p* = .08. Neither the main effect of Condition nor the interaction term was significant, both *p*s > .1.

Thus, our participants felt luckier after wins and unluckier after losses, in both the spinning and no‐spin conditions, with no discernible differences in the objective outcomes between the two versions of the task. Participants reduced their bets following wins, in line with a “gambler's fallacy” account and consistent with Experiment 1.

#### Decomposing neutral outcomes

##### Luck ratings

In the model distinguishing the three types of neutral outcomes (NW vs. NL vs. FM), there was a significant main effect of Condition (see Figure 5A), *χ^2^*(1) = 5.18, *p* < .05, with higher overall luck ratings on the spinning task than the no‐spin task. A possible explanation is that the spinning and decelerating phase in the spin condition made it easier for the participants to generate counterfactuals, thus giving rise to heightened luck‐ratings. The main effect of Outcome Type was significant, *χ^2^*(2) = 86.94, *p* < .001, and qualified by a significant Outcome Type × Condition interaction, *χ^2^*(2) = 51.84, *p* < .001. Simple effects analysis looked at the outcome types in the two conditions separately. In the spinning condition, there was a significant main effect of Outcome Type, *χ^2^*(2) = 89.58, *p* < .001: NL increased self‐perceived luck relative to FM, *b* = 0.87, *t*(102) = 6.01, *p* < .001, while NW decreased luck ratings, *b* = −0.85, *t*(102) = −5.89, *p* < .001. In the no‐spin condition, the main effect of Outcome Type was also significant, albeit with a smaller effect size (*R* ^2^ = .02 in the no‐spin task vs. *R* ^2^ = .18 in the spinning task), *χ^2^*(2) = 13.02, *p* = .0015: NL increased luck ratings compared to FM, *b* = 0.23, *t*(102) = 2.36, *p* < .05, but there was no significant difference between NW and FM, *p* = .19.

##### Betting behavior

There was a significant main effect of Outcome Type (see Figure 5B), *χ^2^*(2) = 8.47, *p* = .01. NW increased bet amount compared to both NL and FM, *b* = 2.16, *t*(202) = 2.27, *p* < .05, and *b* = 2.62, *t*(202) = 2.74, *p* < .01, respectively, but there was no difference between FM and NL, *p* > .1. There was no significant main effect of Condition or interaction term, *p*s > .1.

#### Near outcomes by position

##### Luck ratings

There was a significant three‐way interaction between Condition (spin vs. no‐spinning), Near‐Miss Type (NW vs. NL) and Near‐Miss Position (before vs. after) (see Figure 5C), *χ^2^*(1) = 4.97, *p* < .05. For simple effects analysis, we tested the two‐way interaction between near‐miss type and near‐miss position in the two conditions separately. In the (original) spin condition, there was a significant Near‐Miss Type by Near‐Miss Position interaction, *χ^2^*(1) = 20.55, *p* < .001. For NW, NW‐after were rated as unluckier than NW‐before, *b* = −0.24, *t*(51) = −2.46, *p* = .017. For NL, NL‐after were rated as luckier than NL‐before, *b* = 0.44, *t*(51) = 3.55, *p* < .001. In the no‐spin condition, the interaction between Near‐Miss Type and Near‐Miss Position was only marginally significant, *p* = .07. There was a significant main effect of Near‐Miss Type, *χ^2^*(1) = 9.68, *p* = .0019, such that NL increased luck ratings compared to NW, *b* = 0.36, *t*(51) = 3.26, *p* = .002. The main effect of Near‐Miss Position was not significant, *p* > .1.

##### Betting behavior

There was a significant three‐way Condition by Near‐Miss Type by Near‐Miss Position interaction on the bet amount change (see Figure 5D), *χ^2^*(1) = 5.90, *p* = .015. Considering the two‐way interactions of near‐miss type and near‐miss position in the two conditions separately, there was a reliable interaction in the spinning condition, *χ^2^*(1) = 20.26, *p* < .001. NW‐before increased bet amount change compared to NW‐after, *b* = 7.85, *t*(51) = 3.77, *p* < .001, while NL‐after increase bets relative to NL‐before, *b* = 5.23, *t*(51) = 2.81, *p* < .01. In the no‐spin condition, the near‐miss Type by Position interaction was marginally significant, *p* = .07, and the main effect for Near‐Miss Position was not significant, *p* = .99. The main effect of Near‐Miss Type was significant, *b* = 0.36, *t*(51) = 3.26, *p* < .05, corroborating the finding in the last model that NL increased luck feelings compared to NW.

Thus, the effects of near‐miss position from Experiment 1 were reproduced in the original condition with the spin and deceleration, but were attenuated (to the point of non‐significance) in the no‐spin condition.

#### Subtypes of objective wins

##### Luck ratings

In the model comparing the three types of objective wins (early‐wins vs. clear‐wins vs. late‐wins), there was a significant main effect of Outcome Type (see Figure 6A), *χ^2^*(2) = 36.34, *p* < .001, with late‐wins increasing luck feelings compared to both clear‐wins and early‐wins, *b* = 0.48, *t*(202) = 5.91, *p* < .001, and *b* = 0.39, *t*(202) = 4.86, *p* < .001, respectively. There was no significant main effect of Condition, *χ^2^*(1) = 0.22, *p* > .1, or Condition × Outcome Type interaction, *χ^2^*(2) = 3.17, *p* > .1.

##### Betting behavior

The main effect of Outcome Type was significant (see Figure 6B), *χ^2^*(2) = 9.00, *p* = .01. Both late‐wins and clear‐wins decreased bet amount compared to early‐wins, *b* = −3.27, *t*(202) = −1.84, *p* < .06, and *b* = −5.31, *t*(202) = −2.99, *p* < .01, respectively. Neither the main effect of Condition nor the interaction term was significant, *p* > .1, and *p* = .08, respectively.

#### Subtypes of objective losses

##### Luck ratings

In the model comparing the three subtypes of losses (early‐losses vs. clear‐losses vs. late‐losses), there was a significant main effect of Outcome Type (see Figure 6C), *χ^2^*(2) = 15.69, *p* < .001, and a significant Condition × Outcome Type interaction, *χ^2^*(2) = 11.35, *p* < .01. The main effect of Condition was not significant, *χ^2^*(1) = 0.005, *p* > .1. For simple effects analysis, we looked at differences between loss outcomes in the two conditions separately. In the spinning task, there was a significant main effect of Outcome Type, *χ^2^*(2) = 11.08, *p* < .01: early‐losses were rated as unluckier than both clear‐losses and late‐losses, *b* = −0.44, *t*(102) = −3.35, *p* < .001, and *b* = −0.29, *t*(102) = −2.23, *p* < .05, respectively. In the no‐spin condition, there was also a significant main effect of Outcome Type, *χ^2^*(2) = 15.27, *p* < .001, where both early‐losses and clear‐losses were rated as unluckier than late‐losses, *b* = −0.42, *t*(102) = −3.30, *p* = .001, and *b* = −0.45, *t*(102) = −3.49, *p* < .001, respectively.

##### Betting behavior

There was no significant main effect of Outcome Type or Condition (see Figure 6D), both *p*s > .1. The Outcome Type × Condition interaction was not significant, *p* > .1.

Thus, significant differences emerged between clear‐cut and close‐call outcomes for both objective gains and losses, and these effects were further moderated by the visual display of the motion trajectory during the anticipatory phase.

## General Discussion

Using a wheel‐of‐fortune task, the present study investigated the subjective and behavioral responses to various near events within the framework of counterfactual thinking. Past work on near‐misses has predominantly focused on NW, which are common events in the context of gambling behavior. The present study extended this work by also presenting NL, a logical counterpart of NW, and by further testing for a modulatory role of near‐miss position (i.e. whether the spinner stopped just before or after the missed outcome). In Experiment 1, NL were perceived as much luckier, replicating a previous observation by Wu, van Dijk, and Clark (2015; see also Wohl & Enzle, 2003). Near‐miss position had a differential impact on NW: NW‐after were perceived as unluckier than NW‐before, consistent with our past work using a laboratory slot machine task (Clark et al., 2013). Conversely, NL‐after were rated as luckier compared to NL‐before. In Experiment 2, this interaction between near‐miss type and near‐miss position was corroborated in an independent (and culturally distinct) sample, but the effect of near‐miss position was attenuated in a modified task where the motion trajectory of the spinner during the anticipatory interval was removed. Experiment 1 also included a second decision‐making task measuring affective responses to counterfactual comparisons. Individual differences in “counterfactual potency”—effectively, the degree of affect corresponding to regret and relief—were seen to predict the sensitivity to NW and NL on the wheel‐of‐fortune task.

#### On the counterfactual nature of gambling near‐misses

In Experiment 1, the counterfactual potency index on a standard choice between two lotteries task (Mellers et al., 1999) correlated with the sensitivity to both NW and NL on our gambling task. Elsewhere, the strength or magnitude with which a particular counterfactual is experienced (i.e. counterfactual intensity) has been found to correlate with subsequent affective and behavioral reactions (Sanna & Turley‐Ames, 2000). A recent study characterized counterfactual potency as the multiplicative combination of “if likelihood” (the degree to which the antecedent condition of the counterfactual is perceived to be likely) and “then likelihood” (the perceived conditional likelihood of the outcome of the counterfactual). This measure predicted the extent to which counterfactual thinking influenced judgments of regret, causation and responsibility (Petrocelli et al., 2011). In our gambling task, the NW elicit upward counterfactuals (e.g. “I almost landed on the big one”) whereas NL outcomes induce downward counterfactual (e.g. “I could have gone bankrupt”) (Wohl & Enzle, 2003; Wu et al., 2015). The observed relationship between counterfactual potency and sensitivity to NW and NL provides correlative support for a counterfactual mechanism of processing near‐miss outcomes. Note that in both Sanna and Turley‐Ames (2000) and Petrocelli et al. (2011) who used scenario settings, the index to represent counterfactual strength was derived from the scenario itself to predict its effect upon emotional response. In the present study, the counterfactual potency was derived from an independent task.

The counterfactual account is further strengthened in our data by the interaction effect between near‐miss type (NW vs. NL) and near‐miss position (before vs. after), seen for both luck ratings and bet amount change. For both types of NW, participants compared a neutral outcome with the counterfactual possibility of a (non‐obtained) win. NW‐before implies a trajectory toward the win segment in which the spinner stops just before the win. In this case, the perception of having almost won requires that the participants mentally simulate the spinner moving forward; an additive upward counterfactual (Roese, 1994). For the NW‐after, when participants first experienced the win state and then see the spinner move on to stop in the null segment, the counterfactual requires the mental undoing of the final move, a subtractive upward counterfactual. Our findings suggest that subtractive process makes participants feel unluckier, compared to the additive process. The finding of increased bet following NW‐before compared to NW‐after extended our observation that NW‐before enhanced self‐reported motivation (Clark et al., 2013) by showing a behavioral effect on actual bet adjustment.

The position effect for NL mirrored its effect on NW, such that NL‐after were perceived as luckier compared to NL‐before. Both types of NL made participants compare the neutral outcome with the undesirable loss alternative (i.e. a downward counterfactual). But participants experienced the escape from a major penalty most vividly on the NL‐after outcomes, when the spinner exited the loss zone and entered the safe zone. To experience a downward counterfactual, the participant would need to mentally undo this escape (i.e. a subtractive counterfactual). For NL‐before, the spinner stopped just short of the loss segment, and the participant would need to mentally simulate the extra motion to achieve the downward counterfactual (i.e. an additive counterfactual). As a result, the NL‐after was reliably experienced as luckier compared to NL‐before. The differential effect of additive versus subtractive counterfactual also affected subsequent bet behavior such that NL‐after enhanced bet compared to NL‐before. The interactive effect of near‐miss type by near‐miss position extends Wohl and Enzle (2003, Experiment 2) in which they manipulated near‐miss position but did not find any modulatory effect on either subjective ratings or betting behavior. Taken together, the interactive effect of near‐miss type and near‐miss position is consistent with the established influence of types (upward vs. downward) and structure (additive vs. subtractive) of counterfactual thinking (Roese, 1994).

In Experiment 2, the near‐miss position effect—the dissociable effects of near‐misses occurring in the before versus after positions—was attenuated in the version of the task where the wheel spin and deceleration were no longer visible. This indicates that the visual stimulus of the anticipatory build‐up is necessary to generate the additive versus subtractive thoughts. In contrast, the near‐miss type effect (i.e. NW vs. NL) observed in Experiment 1 was replicated in both conditions in Experiment 2, although was somewhat attenuated in the no‐spin task. Previous research has shown that individuals who are prone to vivid mental imagery tend to have stronger emotional reactivity to counterfactuals (Barlett & Brannon, 2007). These data suggest that the distinction between NW and NL seems to be outcome‐related effects that are not dependent upon the visual input of the motion trajectory.

Our position effect can also be considered within the framework of the reflection and evaluation model (REM) of comparative thinking (see Markman & McMullen, 2003 for a review). The REM proposes that two psychologically distinct models of mental simulation operate during comparative thinking. In *reflection*, an experiential (“as if”) mode of thinking is characterized by vividly simulating that information about the comparison standard is true of, or part of, the self. In evaluation, information about the standard is used as a reference point against which to evaluate one's present standing. Thus, in the case of the NW before outcome, the spinner initially decelerates within the neutral segment, but as the spinner begins to approach the win segment, the participant may mentally simulate the possibility of a win. According to the REM, when attention is focused *only* on the counterfactual outcome (McMullen, 1997), reflective processing triggers an assimilation effect (e.g. “Wow, that was exciting – I nearly won”). In the case of NW after, the participants momentarily expect to win as the spinner is slowing down in a win position. This focuses their attention on winning. At the very end, however, the win is withheld, and this unexpected result focuses their attention on both the NW and the obtained neutral outcome, triggering an *evaluative* contrast effect (e.g. “my loss feels really bad because I almost won”). Thus, the contrast effect for NW after reduced luck ratings compared to the assimilation effect for NW before. In a similar way, the NL before outcome engages reflective processing (i.e. assimilation effect) and thus induces negative affect, whereas the NL after outcome triggers evaluative processing (i.e. contrast effect) to induce positive affect.

#### Other accounts of near‐misses

At this point, it may be appropriate to compare our insights to alternative accounts of near miss effects. One account drawing upon behavioral learning theory is based on frustrative non‐reward. This posits that as goal pursuit is thwarted (e.g. on a NW), it elicits increased reward‐seeking effort (Amsel, 1958). In the case of NW, the salience of the non‐win outcome being proximal to the goal (e.g. the jackpot) induces a negative emotional state (Clark et al., 2012; Dixon, Harrigan, Jarick, Fugelsang, & Sheepy, 2011), which serves to increase the motivation to gamble further, putatively in order to alleviate the aversive state. Frustration theory is compatible with the motivational effects of NW, but is difficult to reconcile with the dissociations between motivational ratings and physiological arousal following NW‐after and NW‐before effects (Clark et al. 2013). Frustration theory also makes no strong predictions concerning NL events. A second contemporary explanation, grounded in the cognitive approach to gambling, is that NW are falsely interpreted as skill acquisition, and thereby foster the “illusion of control” (Billieux, Van der Linden, Khazaal, Zullino, & Clark, 2012; Clark et al., 2013, 2009). In games involving skill, NW outcomes may provide indication of skill acquisition and constitute useful signal of imminent success. Similar to the frustration theory, this learning account is compatible with the motivational effect of NW, but does not readily explain the differential effect of near‐miss position.

By proposing a counterfactual account for near‐misses, we do not seek to argue against these alternative accounts. Near‐misses are psychologically complex events involving conflicting emotional responses and cognitive appraisals, both of which influence subsequent gambling decisions (Clark et al., 2013). Both the frustration and skill acquisition account are compatible with some aspects of the near‐miss findings in the winning context (i.e. NW), but they do not readily explain the position effect, and they do not generate clear predictions for the reaction to a NL, a type of outcome that has received much less empirical attention to date. Recent research using anecdotes of natural disasters suggests that NL influence decision‐making by reducing the future perceived risk of that event happening (Dillon & Tinsley, 2008). The counterfactual account complements these alternative accounts by accommodating both NW and NL findings within one framework.

#### Narrow versus clear objective outcomes

In the present study, we also obtained preliminary evidence showing that people process objective wins and losses differently depending on whether they constitute clear outcomes (i.e. winning or losing by a clear margin) or narrow outcomes (i.e. winning or losing by a narrow margin). Across the two experiments, one type of narrow‐win (the late win) was consistently perceived as luckier, compared to clear‐wins. For the narrow‐wins, people compare the winning outcome with the anticipated, less desirable null outcome (i.e. a downward counterfactual), and this may enhance positive feelings. Some other effects were not consistent across both experiments, and we reserve interpretation of those events until further replications have been attempted. Future research is needed to elucidate the mechanism by which narrow outcomes impact on decision‐making and the effect of the narrow‐miss positions.

#### Relationships between luck ratings and betting behavior adjustment

The present study also demonstrated multiple influences on betting behavior. One line of research has shown that induced feelings of luck increase subsequent risky choice (Darke & Freedman, 1997; Jiang, Cho, & Adaval, 2009; Wohl & Enzle, 2003). Experiencing a lucky event increased self‐reported confidence and betting on a subsequent gamble, and this effect was moderated by personal beliefs in luck conceptualized as a personality trait (Darke & Freedman, 1997). Priming participants with luck‐related concepts also increased their self‐perceived luck and risky financial choices (Jiang et al., 2009). Of more direct relevance to the present study, Wohl and Enzle (2003) found that NL increased luck feelings and increased the amount bet on a subsequent gamble on a separate event. Thus, under some circumstances, temporarily induced lucky states appear to enhance risk taking. Our data speak against this reasoning. As a manipulation check in the present study, objective wins increased self‐perceived luck compared to null outcomes, whereas objective losses decreased self‐perceived luck. However, the bet amount change responded in the opposite direction: objective wins were rated as the luckiest events, but reduced subsequent betting to the greatest extent, and vice versa for objective losses. In our view, these effects are best conceptualized in relation to a gambler's fallacy‐type mechanism; people do not expect successive wins (losses) in a random task, and therefore strategically reduce (increase) their bet to maximize their earnings on the task.

Note that in the present study, after the participant made a bet, the wheel spin was controlled by the computer rather than by the player himself. Perceived control can have important effects in gambling decisions. For instance, individuals have inflated self‐confidence when they can exert irrelevant control over chance event (i.e. illusory control; Langer, Marcus, Roth, & Hall, 1975). Future studies would benefit from testing the modulatory role of perceived control in processing near‐miss outcomes (see Clark et al., 2009).

#### Conclusions

Characterization of the psychological processes that underlie near‐miss experiences not only informs gambling research but also has implications for economic decision‐making in general. The present study demonstrates that individuals who were more susceptible to counterfactual comparisons were also more sensitive to near‐miss outcomes. NW and NL had differential effect upon subjective luck‐feelings and betting behavior, and this was further moderated by near‐miss position, consistent with established influence of upward versus downward and additive versus subtractive thoughts in counterfactual thinking. Critically, the near‐miss position effect depends on the spinning and decelerating effect in the anticipatory phase. Taken together, these date help substantiate the counterfactual account of near‐misses.
